# Clinical, biochemical profile and atherosclerotic cardiovascular risk score (ASCVD) in patients with high HDL cholesterol

**DOI:** 10.1007/s11845-025-03878-8

**Published:** 2025-02-08

**Authors:** Raksha Udupi Mallya, Sudha Vidyasagar, Varashree BS, Cynthia Amrutha Sukumar

**Affiliations:** 1 Department of General Medicine, Kasturba Medical College, Manipal, Manipal Academy of Higher Education, Manipal, Karnataka 576104 India; 2 Department of Biochemistry, Kasturba Medical College, Manipal, Manipal Academy of Higher Education, Manipal, Karnataka 576104 India

**Keywords:** ASCVD, CETP, HDL cholesterol, Lipidology, METs

## Abstract

**Background:**

It was observed that many people in the western coastal belt were found to have a high HDL cholesterol, the cause of which was not known. This study was done to learn about the factors contributing to the high HDL cholesterol in these patients and its effect on ASCVD risk.

**Methods:**

In this prospective, case control study, 150 patients were recruited, of which 63 were cases (patients with high HDL cholesterol), and 87 were controls (patients with normal HDL cholesterol). Details regarding their diet, sea-food consumption, habits, comorbidities, daily activity (using GPAQ questionnaire), and blood reports were collected. ASCVD risk score was calculated using an online ASCVD risk estimator. Blood samples of 96 patients (cases 40, controls 56) was tested for cholesterol esterase transfer protein (CETP) levels using ELISA, and the results were compared.

**Results:**

Patients with high HDL cholesterol were found to be physically more active and had median metabolic equivalent (METs) of 4680 (1200, 8580) compared with controls with median METs of 1680 (720, 5580), P-0.013. Cases had a lower mean BMI 23.09(SD-3.69), than in patients with normal HDL cholesterol with a mean of 24.41(SD-4.01), P-0.04. Cases also had a lower triglyceride level (91(69,118) in cases vs 121 (80,151) in controls, P-0.002. Alcohol and sea food consumption had no role on HDL levels in this study. The median CETP level was lower in patients with high HDL levels, 0.336(0.08, 0.336) versus 1.435(0.061, 2.893) in the control group although not statistically significant. Patients with high HDL cholesterol were found to have a significantly lower median 10-year ASCVD risk score 3.05 (0.6, 8.95), compared with patients with normal HDL 6.45 (2.7,14.2).

**Conclusion:**

Patients with high HDL cholesterol were found to be physically more active, had a lower BMI, a lower triglyceride level, and a lower ASCVD risk compared with controls. They also had a lower CETP level. Further research will be required to determine the normal CETP level in Indian population, their genetic makeup, and whether it has a role in cardiovascular protection.

## Introduction

HDL is a lipoprotein produced by the liver. Its role in reverse cholesterol transport is well known. Low HDL level is a well-known risk factor for ASCVD. In fact, atherogenic dyslipidemia is well known in the Indian population, with metabolic syndrome and diabetes mellitus, and is a cause of increasing coronary artery disease.

Several lifestyle habits like increased physical activity, food habits like Mediterranean diet, and omega 3 fatty acids were found to increased HDL cholesterol levels. Also, moderate amounts of wine consumption were found to increase HDL cholesterol levels. Moreover, there are several genetic disorders that have a role to play in the level of HDL cholesterol, like cholesterol ester transfer protein (CETP), Apo A1 deficiency, and ABCA 1 deficiency. CETP is associated with exchange of triglyceride and cholesterol esters from HDL to other lipoproteins. Deficiency of CETP protein was associated with a high HDL cholesterol. Such high level of HDL cholesterol was noticed in various populations due to loss of variance in CETP. However, it is not known if this high HDL cholesterol is beneficial. Increasing, HDL levels artificially with drugs did not result in a lower cardiovascular risk, as evidenced by several studies in the past. Hence, this strategy to reduce cardiac risk has been abandoned. Therefore, it would be interesting, to see, if these naturally occurring high levels of HDL, are protective for cardiovascular morbidity and mortality.

## Materials and methods

### Study design

This study was a prospective, case control study, conducted over 20 months, from 11 December 2019 to 11 August 2021, in Kasturba Medical College, Manipal. After obtaining informed consent, the required information was collected with the help of a questionnaire.

### Study population

Patients who were in the age group of 18–75 years were included in the study after obtaining consent. Patients with high HDL cholesterol (> 50 mg/dl in male, > 60 mg/dl in female) were identified as cases. Age and sex matched patients with normal cholesterol level were taken as controls. Patients on cholesterol lowering drugs like statin, fibrates, niacin, high intensity exercise, cholesterol lowering diet, uncontrolled diabetes, hypothyroidism, major organ dysfunction, sepsis, clinical evidence of malignancy, steroid/OCP use, and high-risk groups (pregnant, patients living with HIV) were excluded from the study.

Patients attending the out-patient department or admitted in the wards at Kasturba hospital and meeting the inclusion–exclusion criteria were included. Brief history about their socioeconomic data, diet, and data pertaining to the intake of fish and alcohol were collected. For physical activity assessment, Global Physical Activity Questionnaire (GPAQ) was used. History regarding their comorbidities was also collected. Examination of the patient focusing on the blood pressure and anthropometry (height, weight, BMI) were taken.

Fasting lipid profile was measured using Roche Cobas E601 analyzer machine and HDL cholesterol (direct homogenous method using Roche Cobas E601 analyzer machine). LDL cholesterol was then calculated by using Friedewald formula. The standard test values that were already done for the patient were documented. Other investigations like ECG, ECHO, carotid artery Doppler, and lower limb arterial Doppler were noted if already done. The ASCVD risk was calculated using online ASCVD risk estimator.

For CETP measurement, plasma was collected using EDTA as an anticoagulant. It was centrifuged for 15 min at 2000–3000 RPM at 2–8 °C and then stored at – 80 °C. ELISA was performed using human CETP kit by bioassay technology laboratory. A standard curve was plotted by using the average OD for each standard on the *Y* axis against the concentration on the *X* axis. The concentration of CETP protein was obtained using an Optical microplate reader set at 450 nm.

### Statistical analysis

Continuous variables were analyzed by mean and standard deviation for data of normal distribution. Median and interquartile range for non-uniform distribution. Categorical variables were assessed as percentages and frequency. Comparison between groups with categorical parameters was done using Chi-square test. Independent *T*-Test and Mann–Whitney test were used to find out significance between quantitative data. Logistics regression was used to identify factors contributing to ASCVD risk score.

## Results

In this study, 150 subjects were included based on the inclusion and exclusion criteria, of which 63 were cases and 87 were controls. However, the 10-year ASCVD risk was calculated only for 82 patients. The ASCVD risk score could not be calculated for patients < 40 years of age and in patients with atherosclerotic complications. CETP levels were measured for 96 patients using ELISA, of which 40 were cases and 56 were controls.

The clinical profile of patients, diet, sea food consumption, and habits, i.e., smoking and alcohol history, was collected and compared between cases and controls. There was no significant diet preference in either of the groups. The association of alcohol consumption and smoking was also not significant in either of the groups. The baseline characteristics of the cases and the controls are summarized in Table 1 and 2.

**Table 1:** Comparison of clinical profile of patients in the study population

Clinical profile		Number	Cases (%) (*n* = 63)	Controls (%) (*n* = 87)	*P* value
Diet	Vegetarian	35	13 (20.6)	22 (25.3)	0.506
	Non-vegetarian	115	50 (79.4)	65 (74.7)	
Seafood	Yes	104	45 (71.4)	58 (66.7)	0.535
	No	47	18 (28.6)	29 (33.3)	
Alcohol	Current	37	19 (30.2)	18 (20.7)	0.351
	Non-consumers	109	43 (68.3)	66 (75.9)	
	Reformed	4	1 (1.6)	3.4 (3)	
Smoking	Yes	7	3 (4.8)	4 (4.6)	0.962
	No	143	60 (95.2)	83 (95.4)	
Comorbidities					
Hypertension	Yes	33	11 (17.5%)	22 (25.3%)	0.253
Diabetes mellitus	Yes	23	6 (9.5%)	17 (19.5)	0.093

**Table 2:** Comparison of lifestyle and laboratory parameters in cases and controls

Characteristics	Central tendency		*P* value
	Cases	Controls	
Physical activity (MET)	4680 (720, 5580)	1680 (720, 5580)	0.013
BMI	23.09 (3.69)	24.41 (4.01)	0.043
Laboratory values			
LDL	122.52 ± 34.64	132.21 ± 39.24	0.112
Triglycerides	91 (69, 118)	121 (80, 151)	0.002
CETP level	0.34 (0.08, 0.336)	1.44 (0.061, 2.893)	0.956

The information about physical activity of participants was collected using the Global physical activity questionnaire and obtained as Metabolic equivalents. The median of METS was compared in cases and controls that had found it to be statistically significant by Mann- Whitney *U* test. Cases had higher median METS compared to controls. The mean BMI in cases was 23.09 (SD-3.69) and in controls was 24.41 (SD-4.01). Cases had a statistically significant lower BMI than controls by independent sample T test. The median value of TG in cases was 91 (69,118) and in controls was 121 (80,151) and was statistically significant by Mann- Whitney Test. The mean value of LDL in cases was 122.52 ± 34.64 and in controls was 132.21 ± 39.24 but not statistically significant by independent *T* test.

### CETP levels in both groups

**Fig. 1:**
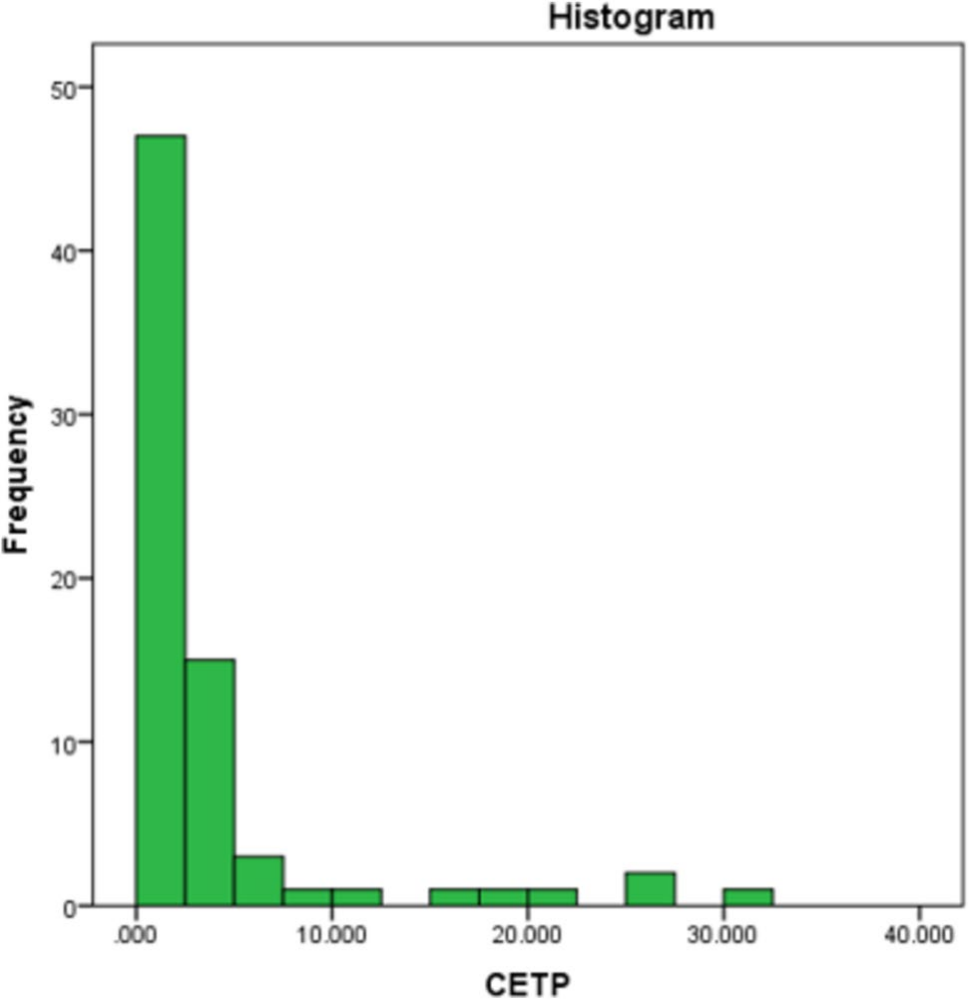
Histogram showing the distribution of CETP level in the study population

**Fig. 2:**
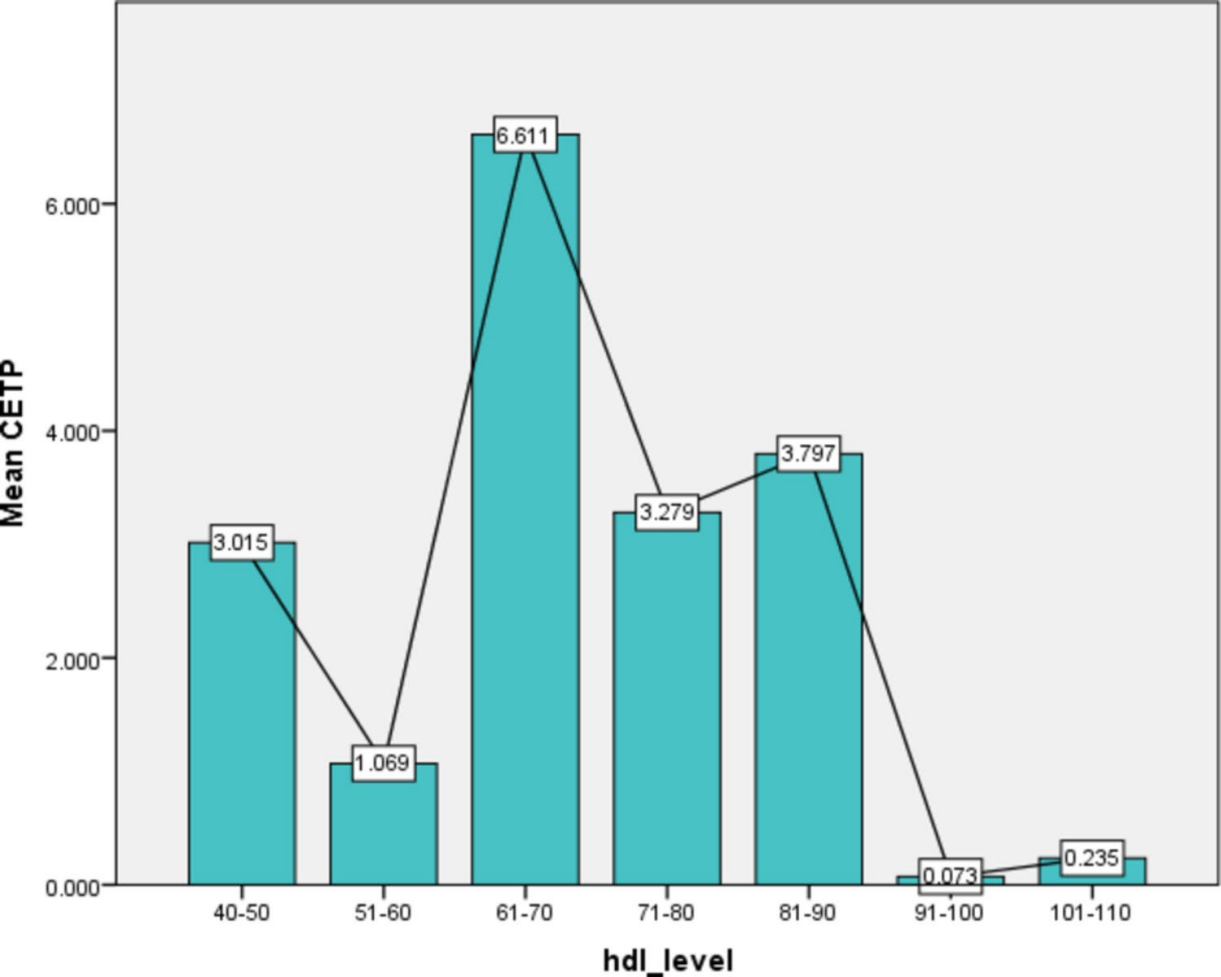
Histogram showing the mean level of CETP in HDL

**Fig. 3:**
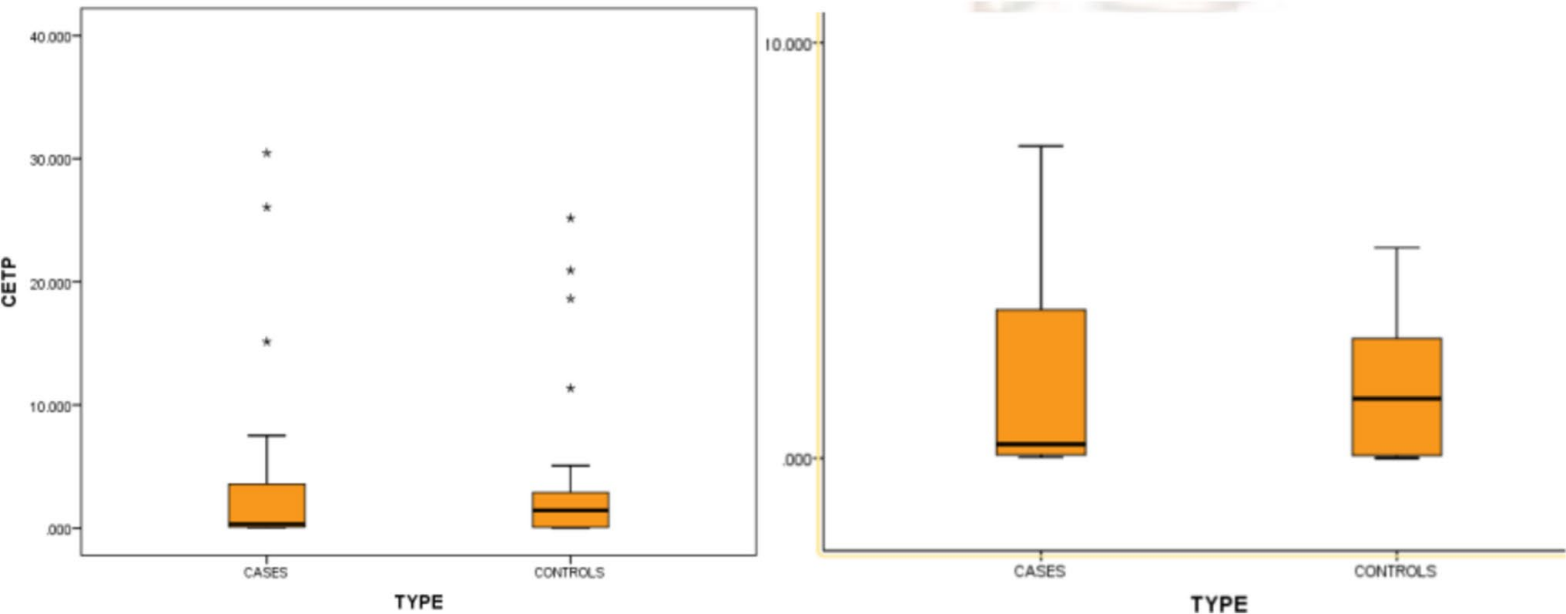
median and IQR of CETP levels in cases and controls

CETP level was calculated in a part of the study population, i.e., in 40 cases and 56 controls. The median CETP level in our study population (as shown in Fig. 1) was found to be 0.437 (0.072,2.962), and distribution was skewed toward the left. But there was no relation between CETP level and HDL levels by correlation analysis. The median CETP levels in cases was 0.336 (0.08, 0.336) and in controls was 1.435 (0.061, 2.893) and was not significant in cases or control by Mann–Whitney test. In our study, we found that the mean CETP level was highest in the patients with HDL levels in the range of 61 to 70 mg/dl, and it decreased on either side (as shown in Fig. 2 and 3).

### ASCVD risk and HDL levels

**Fig. 4:**
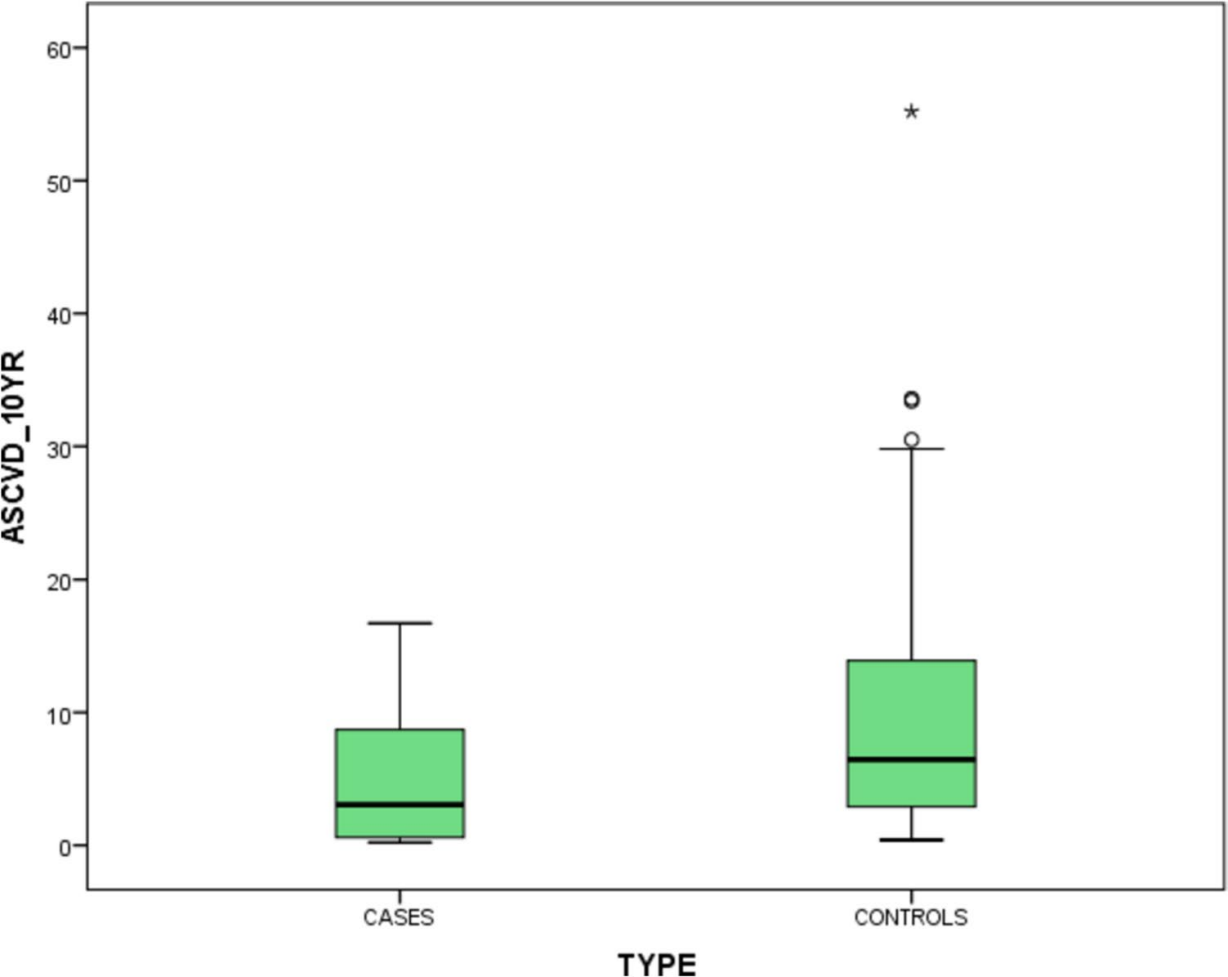
Box plot showing the median and IQR of ASCVD risk scores in cases and controls

The median ASCVD risk score in cases was 3.05 (0.6, 8.95) and in controls was 6.45 (2.7,14.2) as shown in Fig. 4. Cases had a significantly lower ASCVD risk compared with controls, by Mann–Whitney *U* test.

## Discussion

Indian population are known to have atherogenic dyslipidemia, with high triglycerides and low HDL cholesterol levels [1]. Low HDL is a well-known atherosclerotic risk factor. On the contrary, it is not known if high HDL cholesterol is beneficial. It was found that increasing HDL prevented CHD more efficiently than reducing LDL in certain Japanese population [2]. Some of the patients in coastal Karnataka have been observed to have a high HDL cholesterol; the reason for which is not known. It is not known if the high HDL cholesterol is due to the lifestyle, physical activity, alcohol intake, sea food consumption, or if it is due to genetically determined CETP levels in this population. There are not many studies regarding this in our population. In this study, we have analyzed the clinical and biochemical profile and lifestyle of patients with high HDL cholesterol and calculated their CETP levels.

### Relation of physical activity and HDL cholesterol level

It is well known that physical activity produces an increased in HDL cholesterol and is cardioprotective. This effect may be mediated by change in the lipid profile for the better, with increasing HDL cholesterol, and Apo A1, and reduction of triglycerides. The degree to which these lipoprotein levels change, and the extent of exercise needed to produce such a change has been debated [3]. There have been various studies to prove the same. A study by Varady et al. in 2011 compared the effect of alternate day fasting, calorie restriction, and endurance exercise on HDL particle size; in 60 obese patients, he found that HDL cholesterol increased with exercise [4].

Antonio et al., in 2020, through 23 metanalysis, showed that there was great variability with different types of exercises like aerobic, running, and walking; other types of exercises were also included, i.e., weight training exercises and yoga. The frequencies, duration, and intensity of the exercises were also considered. All analysis showed an improvement in HDL cholesterol levels compared with controls [5]. A similar Indian study was done in 2014, across four states (Tamil Nadu, Jharkhand, Maharashtra, and Chandigarh). The physical activity of people was documented based on the GPAQ score; it was found that total cholesterol and triglyceride were less in patients who were highly active and was statistically significant, but there was no difference in HDL levels [6].

In the present study, we interviewed the patients regarding their daily activity during work, travel, and during leisure and converted it into METS. We compared the median METS in case and controls and found that the median value of METS in case was 4680 with interquartile range of (1200, 8580); median value in controls was 1680 (720, 5580). Cases had a higher median METS value compared with controls and was statistically significant. Hence, patients with higher HDL cholesterol were physically more active compared to controls.

### Relation of alcohol and HDL cholesterol level

The “French paradox” postulates that the French have a lower incidence of ischemic heart disease, in spite of a high fat diet containing cheese and meat, due to their consumption of red wine. This was thought to be due to the fact that red wine contains polyphenols and flavonoids which may be responsible for raising HDL cholesterol. It was also found that insulin sensitivity increases after red wine, which has an effect of decreasing triglyceride and increasing HDL [7]. Some studies have also shown that light alcohol consumption was associated with lesser incidence of ischemic heart disease [8].

The possible mechanism for increasing the HDL cholesterol is the presence of polyphenols that have been found to prevent the oxidation of LDL and thus atherosclerosis. They have also been found to improve the endothelial function [9]. In an interventional study by Hansen et al. in 2005, in Denmark, they showed that wine consumption was associated with a significant 11–16% increase in fasting HDL cholesterol [9]. There was an umbrella-shaped correlation between alcohol consumption and HDL levels in a study by Huang et al. in 2005 in China which had enrolled 71,379 people [10].

Vidal et al., in 2010, in Switzerland, did a cross-sectional study and showed that there was an increase in HDL level in alcoholics but not related to genes [11]. Similarly, in a study by Roy et al. in 2010 in India, there was a high HDL level in alcohol users as compared to lifetime abstainers. But he also did not observe an inverse (protective) association between alcohol intake and coronary heart disease [12].

In the present study, we took a history of alcohol consumption in the study subjects. Nineteen (30.2%) cases and 18 (20.7%) controls consumed alcohol, which was not statistically significant. Hence, cases did not have more patients consuming alcohol compared with the controls.

### Relation of diet with HDL cholesterol levels

There have been may theories about foods that improve the lipid profile. Some of these are the Mediterranean diet, fish oils, fatty fish, and olive oil. Many studies have shown that consumption of fatty fish can increase HDL cholesterol levels. It is said that fish oils are rich in omega 3 fatty acids which have an antithrombotic and anti-inflammatory and improve endothelial function [13]. It was also found that traditional Mediterranean diet increased the cholesterol efflux capacity and hence improved HDL cholesterol size and level. When olive oil was added to this diet, it reduced CETP activity and increased HDL ability to esterify cholesterol [14].

The results from a randomized control trial with cross over, by Aadland et al. in Norway, in 2015, showed that lean sea food intake reduces cardiovascular risk factors, i.e., reduced fasting and postprandial triacylglycerol concentration in chylomicrons and VLDL [15]. There was an increase in HDL cholesterol and a decrease in LDL cholesterol and triglycerides in patient consuming fatty fish compared with lean meat in a study done by Hagen et al. in 2016 in Norway [16].

In our study, there was no significant predominance of diet or sea food consumption in cases.

### Relation of BMI with HDL cholesterol Level

HDL levels in obesity have been found to be low. It has been attributed to both an increase in the uptake of HDL2 by adipocytes and an increase in the catabolism of apolipoprotein A-I. There is also a decrease in the conversion of the pre-beta1 subfraction, to pre-beta2 particles [17]. Woudberg et al. in South Africa did a study with a population of 60 women to prove that obesity and ethnicity had a role in HDL cholesterol. It showed that obese women had a lower HDL cholesterol compared with normal weight women [18]. Pascot et al. did a study in Canada in 2001; a sample size was 238 patients to see the relevance of small HDL cholesterol with abdominal obesity. The study showed that obese individuals had a reduced concentration and size of HDL cholesterol [19]. In a case control study in India by Bora et al., 190 patients were taken, and it was found that people who were overweight or generalized obesity had a significantly greater odds of developing decreased HDL [20].

In the present study, we measured BMI of all participants; controls had a higher average BMI mean 24.41 $\pm$ 4.01 compared with cases 23.09 $\pm$ 3.69, by independent *t* test; *P* value was 0.043. Patients with higher HDL had a lower BMI compared with controls.

### Hypertension and HDL cholesterol levels

In Japanese individuals, it was found that there was an association between HDL levels and hypertension. The exact mechanism of the same is not known [21]. In 2017, Cho et al. did a study in Korea on 4662 patients, and it was found that elevated DBP and SBP were associated with a decrease in HDL-C [22].

In the present study, we found that 1.7% cases were hypertensive, and 25.3% controls were hypertensive. There was no significant predominance of hypertension in cases or controls.

### Diabetes mellitus and HDL cholesterol levels

In T2DM, insulin resistance leads to hypertriglyceridemia, hyperglycemia, and hyperinsulinemia. There is accelerated lipolysis due to insulin resistance and an increase in free fatty acids. As a result, CETP activity also increases, leading to increase in triglycerides in HDL and reduction in the level of triglycerides. Hepatic lipase leads to accelerated clearance of the HDL cholesterol in this scenario.

Many studies have been done to see the relation of diabetes with levels. In 2007, Okin et al., in the USA, found that lower HDL cholesterol was more strongly associated with an increased risk of diabetes mellitus in a study population of 7445 people in USA [23]. Similarly, in 2012, Masaram et al. found that in 100 Indians, the HDLC level decreased in both types of diabetes as compared to the controls [24].

In the present study, we compared the number of diabetics in cases and controls. We found that there was no significant difference in the number of diabetics in cases and controls.

### Comparison of CETP levels

CETP is a protein produced in the liver and is present over the HDL cholesterol. Their levels and activity are genetically determined. Their function is to shuttle triglycerides from apo B containing lipoproteins to HDL and the transfer cholesterol esters from HDL to apo B containing lipoproteins. This triglyceride containing HDL cholesterol is rapidly cleared by the liver, leading to reduced HDL levels. It was found that some individuals had a genetically low CETP level leading to increased HDL cholesterol. But it is not clear whether this is beneficial or not [25]. Multiple drugs were developed to inhibit CETP protein level and increase the HDL cholesterol.

The CETP levels was measured in a study in Japan by Zhuang et al. in 2001. The median CETP level in their population was 1.3. The mean was 1 in male and 1.95 in female [26]. In a case control study by Devi et al. on 50 cases (patients with angiogram proven atherosclerosis) and 50 controls (with normal angiogram), in New Delhi, the mean CETP level was 0.37 $\pm$ 0.21 in the normal angiogram group [27].

In our study, we collected blood samples of 96 patients, of which 56 were cases and 40 were controls. We calculated the CETP protein level with the help of a CETP ELISA kit by bioassay technology laboratory. The CETP values were calculated by reading the optical microplate reader. The values were obtained for 79 samples. The median value of CETP in our study subjects was 0.44 (0.07, 2.96).

The values were skewed toward the lower side, which was similar to the above-mentioned study by Zhuang et al. Very few individuals had a very high CETP level. It was also found that the mean CETP level was higher in patients with HDL level in the range of 60–70 mg/dl. Similar results were also documented in study in Japan; the reason for which is not known [28]. The median CETP level in cases was 0.336 (0.08, 0.336) and in controls was 1.435 (0.061, 2.893). Cases had a lower CETP level compared to controls, but it was not significant by Mann–Whitney *U* test; *P* value was 0.956.

### HDL and ASCVD risk scores

It is established that low levels of HDL cholesterol point to an unfavorable lipid profile, increasing the prevalence of ischemic heart disease and its consequences. Metabolic syndrome includes low HDL, and it is counted as a marker of insulin resistance and visceral obesity, both of which contribute to cardiac disease [29].

However, what is often debated is if this is a causal relationship, between low HDL and CV risk, or HDL is only a risk marker, like a biomarker of the presence of atherosclerosis [30]. Further, it has been observed that higher levels of HDL cholesterol 90 mg/dl in men and 75 mg/dl in women did not show any benefit [31].

In our study, we calculated the 10-year ASCVD risks score in cases and in controls using the ASCVD risk estimator. The median value in cases was 3.05 (0.6, 8.95) and in controls was 6.45 (2.7,14.2). It was significantly higher in controls by Mann–Whitney *U* test; *P* value was 0.001. Hence, cases had a higher ASCVD risk compared to controls.

It may be interesting to note that we found higher ASCVD score among our cases (who had high HDL cholesterol). This could be attributed to other factors involved in ASCVD score (blood pressure, total cholesterol, hypertension, and diabetes) which could have affected the final ASCVD score in the cases and controls despite high HDL among the cases.

### Strengths and limitations of the study

Our study is one of the few studies done to measure CETP levels in the Indian population, and this is a major strength. However, the normal levels of CETP protein in Indians is not known, as this area has not been explored well in Indian population.

As this is cross sectional, the long-term consequences are unclear, and this may need follow-up.

There are not many studies evaluating the cardiovascular risk benefit of high HDL levels, and also there are very few studies analyzing the various factors like diet, alcohol, and sea food consumption affecting the level of HDL cholesterol in India.

## Data Availability

Available on contacting the corresponding author.
